# Clinical manifestations and pathological correlation of immunoglobulin A nephropathy in children

**DOI:** 10.1186/s12882-022-03002-3

**Published:** 2022-11-16

**Authors:** Karnchanit Sausukpaiboon, Sunee Panombualert, Suwannee Wisanuyotin, Anucha Puapairoj, Piyanan Suparattanagool, Leelawadee Techasatian, Nattakarn Tantawarak

**Affiliations:** 1 Department of Pediatrics, Pediatric Nephrology Division, Khon Kaen University, Khon Kaen, 40002 Thailand; 2 Department of Pathology, Khon Kaen University, Khon Kaen, Thailand; 3 Clinical Epidemiology Unit, Khon Kaen University, Khon Kaen, Thailand

**Keywords:** IgA nephropathy, Children, Pathology, Clinical manifestation

## Abstract

**Background:**

IgA nephropathy in children has various clinical manifestations. Kidney biopsy is a gold standard for diagnosis by using Oxford classification 2016 with few studies about the correlation between clinical and pathology manifestations. This study aims to find these correlations at the time of diagnosis and during short-term follow-up.

**Method:**

In this retrospective cohort study, 47 pediatric patients who underwent renal biopsy from 2010 to 2021 in Thailand, were included. Oxford classification 2016 has been used to score patients’ pathology. Univariate and multivariate associations have been used for correlation between clinical and pathologic parameters.

**Results:**

The most common clinical manifestations were microscopic hematuria and proteinuria. There were 68% of children with mesangial hypercellularity (M1), 42% with segmental glomerulosclerosis (S1), 25% with moderate to severe crescent (C1/C2), 23% with endocapillary hypercellularity (E1), and 14% with moderate to a severe tubular atrophy/interstitial fibrosis (T1/T2). Microscopic hematuria was strongly associated with mesangial hypercellularity (M1) OR 7.14 (95%CI 1.83 – 27.88, p-value 0.005) and hypertension was strongly associated with segmental glomerulosclerosis (S1) adjusted OR 7.87 (95%CI 1.65 – 37.59, p-value 0.01). Intensive treatment was used more in the patients with tubular atrophy/interstitial fibrosis lesion on renal biopsy than other lesions from MEST-C scores OR 4.98 (95%CI 1.17–21.24, p-value 0.03). Furthermore, pulse methylprednisolone and cyclophosphamide were used in patients with crescentic lesions significantly than other lesions with OR 15.5 (95%CI 3.16- 75.93, p-value 0.001) and OR 5.75 (95%CI 1.31–25.29, p-value 0.021), respectively.

**Conclusion:**

Tubular atrophy/interstitial fibrosis and crescent lesions were correlated to intensive treatment in short-term outcomes.

## Introduction

Immunoglobulin A nephropathy (IgA nephropathy) or Berger’s disease is a common cause of primary glomerulonephritis [1]. IgA nephropathy occurs with the greatest frequency in East Asian individuals especially in Japan due to the urinalysis screening in child health policy [2]. There are various clinical manifestations of IgA nephropathy in children. Microscopic hematuria and/or proteinuria are common in urine screening. Almost half of the patients present with one or recurrent episodes of gross hematuria, that often happens after an upper respiratory tract infection. Less than 10 percent present with nephrotic syndrome or an acute, rapidly progressive glomerulonephritis characterized by edema, hypertension, and renal insufficiency as well as hematuria.

The diagnosis of IgA nephropathy can be confirmed only by renal biopsy with immunofluorescence studies for IgA deposits. The advanced clinical presentations such as a decreased glomerular filtration rate (GFR) and increased proteinuria, are commonly associated with chronic pathological features like glomerulosclerosis, tubulointerstitial inflammation, tubular atrophy, and interstitial fibrosis. Oxford classification has been developed for pathologic features of IgA nephropathy in terms of MEST-C scores [3].

Nowadays, combined clinicopathologic analysis information is used to predict a renal outcome and is important for management decisions. T score reflects the stage of the disease at the time of biopsy; those patients with more advanced chronic damage have a shorter time to end-stage renal disease (ESRD) [3, 4]. As well as active cellular lesions, M and E scores were also associated with the deterioration rate of renal function. Crescents were also found to be predictive of poor renal outcomes and strongly associated with subsequent use of immunosuppression [3, 4].

However, the previous study stated only the pathological correlation with long-term renal survival. There was no current research study to determine the clinicopathological correlation at diagnosis and during short-term follow-up. This study aims to find the correlation between the presenting of symptoms and the severity of pathological findings. We assume that these results would be useful for early treatment decisions.

## Methods

### Study design, data collection, and population

A total of 50 children aged less than 18 years old who were diagnosed with IgA nephropathy retrospectively from January 2010 to April 2021, were included. All patients underwent renal biopsy in a tertiary care hospital in Thailand with a definite diagnosis. We excluded 3 patients due to incomplete pathological data, finally, 47 patients remained in our study.

Demographic data consists of gender, date of birth, age at biopsy. The clinical presentations were recorded at the time of biopsy and during follow-up at 6, 12, and 24 months after treatment included systolic blood pressure (SBP), diastolic blood pressure (DBP), mean arterial pressure (MAP), serum creatinine, estimated glomerular filtration rate, microscopic hematuria, gross hematuria, significant proteinuria, nephrotic range proteinuria, edema, acute kidney injury, rapidly progressive glomerulonephritis, and chronic kidney disease. Pathologic parameters in MEST-C score [3] and treatment modalities were recorded including angiotensin-converting enzyme, corticosteroid, and other immunosuppressants. All MEST-C scores were determined by the same pathologist for persistence validation.

## Study definitions

- Significant proteinuria, defined by urine protein creatinine ratio ≥ 0.5 mg/mg in children 6 months to 2 years old, ≥ 0.2 mg/mg in children age more than 2 years old.
- Nephrotic range proteinuria, defined by urine protein creatinine ratio more than 2 mg/mg.
- Acute kidney injury;(KDIGO) [5]

**Table:** 

Stage	Serum creatinine (SCr)	Urine output
1	Increase to 1.5 to 1.9 times baseline, or increase of ≥ 0.3 mg/dL	< 0.5 mL/kg/ hour for 6 to 12 h
2	Increase to 2 to 2.9 times baseline	< 0.5 mL/kg/hour for ≥ 12 h
3	Increase greater than 3 times baseline, or SCr ≥ 4 mg/dL, or initiation of renal replacement, or eGFR < 35 mL/min per 1.73 m^2^ (< 18 years)	< 0.3 mL/kg/hour for ≥ 24 h, or Anuria for ≥ 12 h

-Oxford classification 2016 [3]

- Mesangial hypercellularity (M), defined as more than four mesangial cells in any mesangial area of a glomerulus: M0 is mesangial cellularity in < 50% of glomeruli; M1 ≥ 50%
- Endocapillary hypercellularity (E), defined as hypercellularity due to an increased number of cells within glomerular capillary lumina: E0 is an absence of hypercellularity; E1 is hypercellularity in any glomeruli
- Segmental glomerulosclerosis (S), defined as adhesion or sclerosis (obliteration of capillary lumina by matrix) in part of but not the whole glomerular tuft: S0 is an absence of segmental glomerulosclerosis, S1 is the presence of segmental glomerulosclerosis in any glomerulus
- Tubular atrophy/interstitial fibrosis (T), defined as the estimated percentage of cortical area showing tubular atrophy or interstitial fibrosis, whichever is greater: T0 is 0–25%; T1 is 25–50%; T2 is > 50%
- Crescents (C): C0 is no crescents; C1 is crescents in less than one-fourth of glomeruli, and C2 is crescents in over one-fourth of glomeruli.

### Data analysis

Data were analyzed using the STATA software program version 10.1. The continuous data were demonstrated using mean and standard deviation. Non-normal distribution was demonstrated using a median and interquartile range. Independent sample T-test or Mann–Whitney U-test was used to compare continuous data, and Pearson chi-square or Fisher’s exact test was used for categorical data. Univariate and multivariate associations between clinical manifestation of IgA nephropathy and pathological parameters and renal survival were performed using the Cox regression analysis and Cox proportional hazards modeling. Renal survival, estimated by a 50% reduction in renal function was used as the secondary outcome. Renal survival curve was measured from the time of biopsy and was examined by the time to event analysis using the Kaplan–Meier method, and quality of survivor functions was examined by the log-rank testing.

## Result

**Fig. 1:**
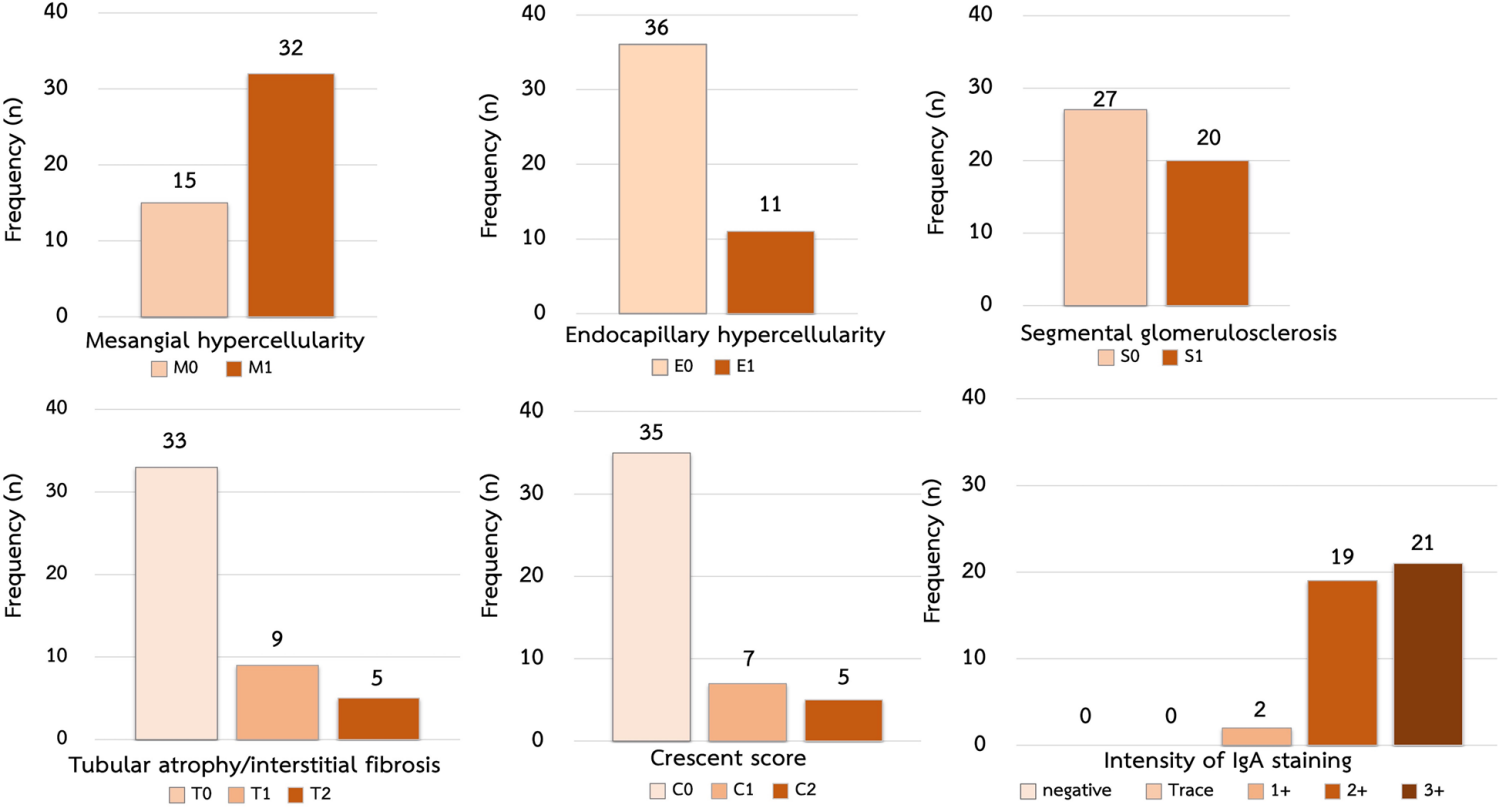
Renal pathological finding at diagnosis categorized by MEST-C scores

A total of 47 pediatric patients were included in this study. Male children were in predominance (59.6%). The mean age was 13 ± 3.4 years old. Median serum creatinine was 0.6 mg/dL (0.5–1.5 mg/dL) and urine protein creatinine ratio at diagnosis was 1.74 (0.7–6.4 mg/mg). The demographic data were shown in Table 1. The most common clinical manifestation was microscopic hematuria. All patients underwent renal biopsy for diagnosis and it was found that mesangial hypercellularity was the most common pathological finding in our study. The pathological finding by MEST-C scores at diagnosis were shown in Fig. 1. There were 32 patients (68%) with mesangial hypercellularity (M1), 20 patients (42%) with segmental glomerulosclerosis (S1), 14 patients (29%) with moderate to severe tubular atrophy (T1/T2), 12 patients (25%) with moderate to severe crescent formation (C1/C2), and 11 patients (23%) with endocapillary proliferation (E1).

**Table 1:** Demographic characteristics of patients

**Clinical characteristic at diagnosis**	**Total** ***n* = 47**
• Male n (%)	28(59.6)
• Mean age at diagnosis (year) (min, max)	13.0 ± 3.4(6,18.6)
• Mean weight (Kg) (min, max)	48.0 ± 20.2(17,113.3)
• Mean height (cm) (min, max)	146.9 ± 17.3(117.5,179)
• Mean arterial pressure (mmHg) (median, IQR)	88.6(82.3,103.0)
• Serum creatinine (mg/dL) (median, IQR)	0.6(0.5,1.5)
• Estimate glomerular filtration rate (mL/min/1.73m^2^) (median, IQR)	97.8(46.9,112.8)
• Urine protein creatinine ratio (mg/mg) (median, IQR)	1.74(0.7,6.4)
**Clinical manifestations at diagnosis**	**n(%)**
• Microscopic hematuria	30(63.8)
• Significant proteinuria	22(46.8)
• Nephrotic range proteinuria	22(46.8)
• Hypertension	21(44.6)
• Edema	20(42.5)
• AKI/RPGN	14(29.7)
• Gross hematuria	10(21.2)
**Treatment after diagnosis**	**n(%)**
• Prednisolone	36(76.6)
• Angiotensin converting enzyme inhibitors	30(63.8)
• Cyclophosphamide	21(44.7)
• Pulse methylprednisolone	12(25.5)
• Mycophenolate mofetil	7(14.9)
• Calcineurin inhibitors	1(2.1)

Data are expressed as number (%), median and interquartile range or mean ± standard deviation

*AKI* Acute kidney injury, *IQR* Interquartile range, *RPGN* Rapidly progressive glomerulonephritis

After IgA nephropathy was diagnosed, prednisolone and angiotensin-converting enzyme inhibitors were mainly used in our patients 76.6% and 63.8%, respectively. Other immunosuppressive drugs were used in some patients such as cyclophosphamide(44.7%), mycophenolate mofetil(14.9%), and calcineurin inhibitors(2.1%) (Table 1).

During follow-up time after treatment at 6,12, and 24 months, there was no statistical change of median serum creatinine as well as the median estimated glomerular filtration rate (Table 2). However, there were eight patients had eGFR less than 60 mL/min/1.73 m^2^ at 6 months. At 24 months of follow up, four patients’ eGFR got better after treatment, but there were four patients still had eGFR less than 60 mL/min/1.73 m^2^ and all of them need dialysis. Whereas edema and gross hematuria significantly improved. Less frequent manifestations such as acute kidney injury and rapidly progressive glomerulonephritis (RPGN) were found in 14 patients. The rates of hypertension, microscopic hematuria, and nephrotic range proteinuria were found the higher rate at 24 months compared to 6 and 12 months in all patients (Table 2). The mean time to be free from nephrotic range proteinuria was 22.75 months (95%CI 21.38 – 24.13).

**Table 2:** Clinical outcome at 6, 12, and 24 months after treatment

Clinical	At diagnosis (*n* = 47)	6 months (*n* = 37)	12 months (*n* = 33)	24 months (*n* = 25)
Median serum creatinine (mg/dL) (IQR)	0.60 (0.5,1.5)	0.60 (0.48,1.02)	0.60 (0.45,0.74)	0.63 (0.5,1.0)
Median eGFR (mL/min/1.73m^2^)	97.8	105.7	104.7	98.8
Hypertension (%)	44.7	27	18.2	28
Edema (%)	42.6	13.5	6.1	8
Microscopic hematuria (%)	63.8	45.9	42.4	52
Gross hematuria (%)	21.3	0	0	0
Significant proteinuria (%)	46.8	70.3	63.6	48
Nephrotic range proteinuria (%)	46.8	16.2	12.1	24
AKI/Rapidly progressive glomerulonephritis (%)	29.8	2.7	0	0
Chronic kidney disease (%)	0	18.9	15.2	24

Data are expressed as number (%) or median and interquartile range

*AKI* Acute kidney injury, *IQR* Interquartile range, *eGFR* Estimated glomerular filtration rate

There was only 1 patient who received deceased donor kidney transplantation. The mortality rate in our study was 2.7%, from one patient who developed septic shock, acute kidney injury, and respiratory failure after treatment with prednisolone and oral cyclophosphamide.

In the correlation between clinical manifestations and pathological findings, we found that microscopic hematuria was strongly associated with mesangial hypercellularity (M1) OR 7.14 (95% CI 1.83–27.88, p-value 0.005). Hypertension was also strongly associated with segmental glomerulosclerosis (S1) OR 7.87 (95%CI 1.65–37.59, p-value 0.01). In contrast, significant proteinuria and nephrotic range proteinuria were not correlated with tubular atrophy/interstitial fibrosis (T1/T2) as shown in Table 3. We also found that all four patients who had chronic kidney disease and required dialysis had global glomerulosclerosis 20–80% from the kidney biopsies at diagnosis.

**Table 3:** Correlation between clinical manifestations and pathological findings categorized by MEST-C scores

**Mesangial hypercellularity**	**M0** **(*n*** **= 15)**	**M1** **(*n*** **= 32)**	**Odds ratio (95%CI)**	***p*-value**	**Adjusted odds ratio (95%CI)**	***p*-value**
**Hypertension**	4 (26.67)	17 (53.13)	3.12 (0.82 – 11.89)	0.096	1.75 (0.37 – 8.23)	0.476
**Edema**	5 (33.33)	15 (46.88)	1.76 (0.49 – 6.34)	0.384		
**Microscopic hematuria**	5 (33.33)	25 (78.13)	7.14 (1.83 – 27.88)	0.005		
**Gross hematuria**	2 (13.33)	8 (25)	2.17 (0.40 – 11.74)	0.370		
**Significant proteinuria**	9 (60)	13 (40.63)	0.46 (0.13 – 1.59)	0.219	0.44 (0.12 – 1.68)	0.232
**Nephrotic range proteinuria**	5 (33.33)	17 (53.13)	2.27 (0.63 – 8.14)	0.210		
**AKI/RPGN**	2 (13.33)	12 (37.50)	3.90 (0.75 – 20.34)	0.106	3.12 (0.47 – 20.85)	0.240
**Median MAP (IQR)**	88.6 (86.3—92)	90.8 (82.3 – 106.15)	1.03 (0.99 – 1.08)	0.171		
**Median UPCR (IQR)**	0.99(0.7 – 2.1)	2.6 (0.98 – 6.65)	1.12 (0.92 – 1.35)	0.260		
**Endocapillary hypercellularity**	**E0 (*n* = 36)**	**E1 (*n* = 11)**	**Odds ratio (95%CI)**	***p*-value**	**Adjusted odds ratio (95%CI)**	***p*-value**
**Hypertension**	13 (36.11)	8 (72.73)	4.72 (1.06 – 20.96)	0.041	2.22 (0.20 – 24.52)	0.517
**Edema**	12 (33.33)	8 (72.73)	5.33 (1.19 – 23.83)	0.028	3.45 (0.45 – 26.14)	0.231
**Microscopic hematuria**	19 (52.78)	11 (100)	NA	NA		
**Gross hematuria**	10 (27.78)	-	NA	NA		
**Significant proteinuria**	21 (58.33)	1 (9.09)	0.07 (0.01 – 0.62)	0.017	0.02 (0.001 – 0.93)	0.046
**Nephrotic range proteinuria**	13 (36.11)	9 (81.82)	7.96 (1.49 – 42.56)	0.015	0.21 (0.008 – 5.35)	0.344
**AKI/RPGN**	9 (25)	5 (45.45)	2.5 (0.61 – 10.20)	0.202		
**Median MAP (IQR)**	88.15 (81.95 – 95.6)	103 (90.6—118)	1.04 (1.00 – 1.08)	0.052	1.03 (0.97 – 1.09)	0.354
**Median UPCR (IQR)**	1.46 (0.7 – 4.66)	6.11 (3.6 – 9.1)	1.13 (0.97 – 1.31)	0.105		
**Segmental glomerulosclerosis**	**S0 (*n* = 27)**	**S1 (*n* = 20)**	**Odds ratio (95%CI)**	***p*-value**	**Adjusted odds ratio (95%CI)**	***p*-value**
**Hypertension**	7 (25.93)	14 (70)	6.67 (1.84 – 24.14)	0.004	7.87 (1.65 – 37.59)	0.010
**Edema**	10 (37.04)	10 (50)	1.70 (0.53 – 5.50)	0.376		
**Microscopic hematuria**	15 (55.56)	15 (75)	2.40 (0.68 – 8.50)	0.175		
**Gross hematuria**	8 (29.63)	2 (10)	0.26 (0.05 – 1.41)	0.120		
**Significant proteinuria**	12 (44.44)	10 (50)	1.25 (0.39 – 3.99)	0.706		
**Nephrotic range proteinuria**	12 (44.44)	10 (50)	1.25 (0.39 – 3.99)	0.706		
**AKI/RPGN**	6 (22.22)	8 (40)	2.33 (0.65 – 8.34)	0.192	0.72 (0.14 – 3.81)	0.704
**Mean MAP (SD)**	89.19 (13.62)	100.39 (20.76)	1.04 (1.00 – 1.08)	0.044		
**Median UPCR (IQR)**	1.25 (0.7 – 5.5)	2.6 (1.17 – 6.80)	0.99 (0.86 – 1.13)	0.835		
**Tubular atrophy/interstitial fibrosis**	**T0** **(*n* = 33)**	**T1/T2** **(*n* = 14)**	**Odds ratio (95%CI)**	***p*-value**	**Adjusted odds ratio (95%CI)**	***p*-value**
**Hypertension**	11 (33.33)	10 (71.43)	5 (1.27 – 19.62)	0.021	1.86 (0.27 – 12.86)	0.527
**Edema**	13 (39.39)	7 (50)	1.54 (0.44 – 5.42)	0.502		
**Microscopic hematuria**	18 (54.55)	12 (85.71)	5 (0.96 – 25.94)	0.055		
**Gross hematuria**	9 (27.27)	1 (7.14)	0.21 (0.02 – 1.80)	0.153		
**Significant proteinuria**	19 (57.58)	3 (21.43)	0.20 (0.05 – 0.86)	0.030		
**Nephrotic range proteinuria**	12 (36.36)	10 (71.43)	4.38 (1.12 – 17.03)	0.033	3.33 (0.77 – 14.32)	0.106
**AKI/RPGN**	7 (21.21)	7 (50)	3.71 (0.97 – 14.18)	0.055		
**Median MAP (IQR)**	88 (81.6—92)	102.15 (90.6—120)	1.05 (1.01 – 1.09)	0.020	1.03 (0.98 – 1.09)	0.255
**Median UPCR (IQR)**	1.36 (0.70 – 3.38)	6.28 (3.1 – 7.33)	1.15 (0.98 – 1.34)	0.081		
**Crescent**	**C0** **(*n* = 35)**	**C1/C2** **(*n* = 12)**	**Odds ratio (95%CI)**	***p*-value**	**Adjusted odds ratio (95%CI)**	***p*-value**
**Hypertension**	11 (31.43)	10 (83.33)	10.91 (2.04 – 58.39)	0.005	2.42 (0.23 – 25.25)	0.461
**Edema**	12 (34.29)	8 (66.67)	3.83 (0.96 – 15.37)	0.058		
**Microscopic hematuria**	22 (62.86)	8 (66.67)	1.18 (0.30 – 4.71)	0.813		
**Gross hematuria**	6 (17.14)	4 (33.33)	2.42 (0.55 – 10.70)	0.245		
**Significant proteinuria**	19 (54.29)	3 (25)	0.28 (0.06 – 1.22)	0.089		
**Nephrotic range proteinuria**	13 (37.14)	9 (75)	5.08 (1.16 – 22.20)	0.031	4.31 (0.72 – 25.90)	0.111
**AKI/RPGN**	7 (20)	7 (58.33)	5.6 (1.36 – 23.06)	0.017	1.92 (0.26 – 14.25)	0.522
**Mean MAP (SD)**	88.83 (11.51)	108.91 (24.02)	1.07 (1.02 – 1.13)	0.004	1.04 (0.98 – 1.11)	0.223
**Median UPCR (IQR)**	1.18 (0.69 – 3.6)	6.27 (3.83 – 7.33)	1.14 (0.98 – 1.33)	0.082		

Data are expressed as number (%), median and interquartile range or mean ± standard deviation

*AKI* Acute kidney injury, *eGFR* Estimated glomerular filtration rate, *IQR* Interquartile range, *MAP* Mean arterial pressure, *RPGN* Rapidly progressive glomerulonephritis, *SD* Standard deviation, *UPCR* Urine protein creatinine ratio

**Fig. 2:**
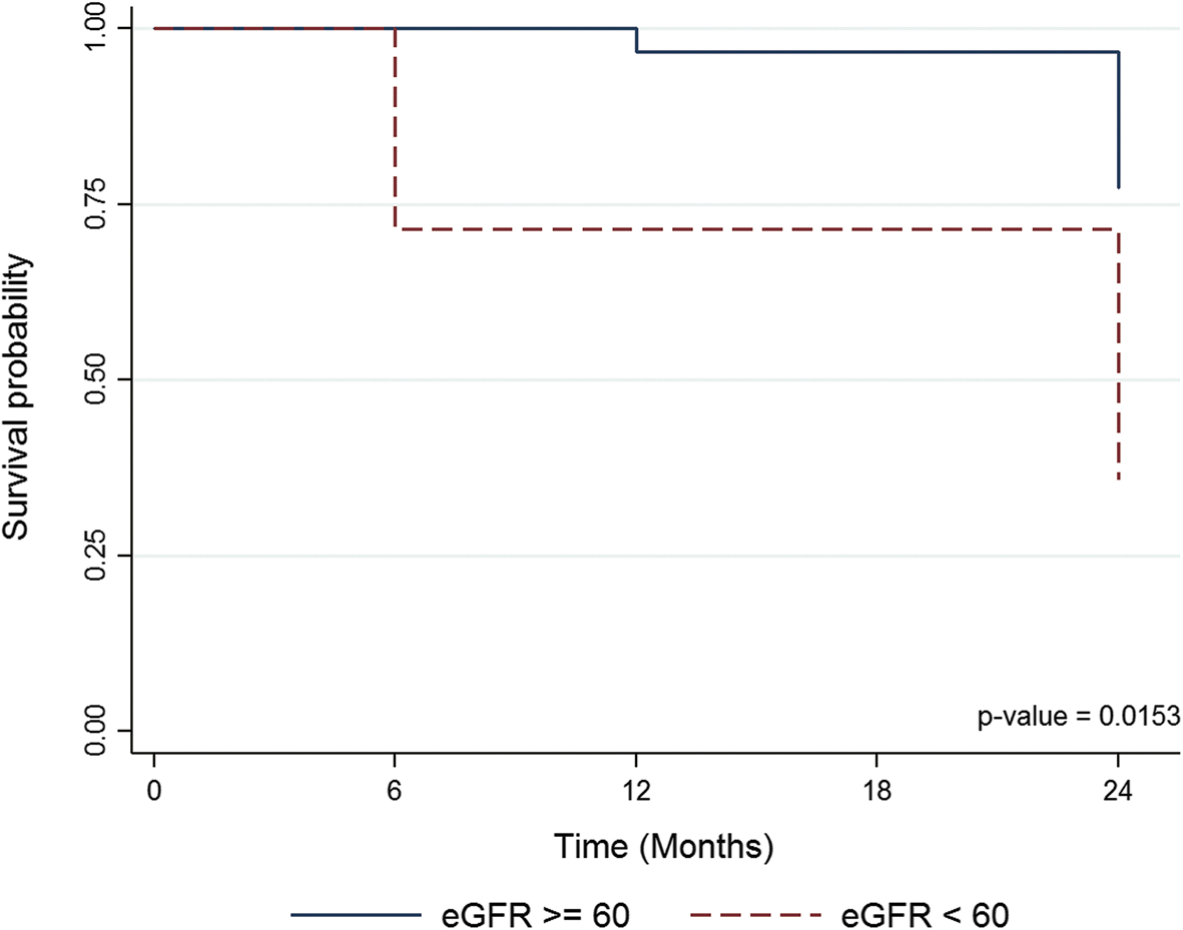
Renal survival according to eGFR at 6, 12, and 24 months. eGFR, estimated glomerular filtration rate

The mean time of renal survival between the patients who had eGFR less than 60 and those with eGFR greater or equal 60 mL/min/1.73m2 was significantly different, 18.9 vs. 23.6 months (p-value 0.015) as shown in Fig. 2.

In the treatment and pathological findings, we also found correlation between the patients who received pulse methylprednisolone, had tubular atrophy/interstitial fibrosis (T1/T2) on renal biopsy greater than other treatments without pulse methylprednisolone (OR 9.67, 95%CI 2.18—42.79, p-value 0.003). The patients who received cyclophosphamide, mycophenolate mofetil, or cyclosporin A, also had tubular atrophy/interstitial fibrosis lesion (T1/T2) more than those who did not receive OR 4.98 (95%CI 1.17–21.24, p-value 0.03). This result reflects that intensive treatment was used more in the patients with tubular atrophy/interstitial fibrosis lesion on renal biopsy than other lesions from MEST-C scores.

Furthermore, pulse methylprednisolone and cyclophosphamide were used significantly more in patients with crescentic lesions than in other lesions with OR 15.5 (95%CI 3.16- 75.93, p-value 0.001) and OR 5.75 (95%CI 1.31–25.29, p-value 0.021), respectively.

## Discussion

Patients with IgA nephropathy have various clinical manifestations from mild symptoms such as microscopic hematuria, proteinuria, edema, hypertension to severe symptoms like rapidly progressive glomerulonephritis that need renal replacement therapy [6–8]. Microscopic hematuria and proteinuria were the most common manifestations of IgA nephropathy in Japanese patients because of the routine urinalysis screening in Japan [6]. In Korea, IgA nephropathy was found as the second most common pathological finding in children who underwent renal biopsy with clinical hematuria [9]. Although in Thailand, we do not have routine urinalysis screening, microscopic hematuria was still the most common clinical manifestation in IgA nephropathy.

The main drugs of choice for IgA nephropathy are angiotensin-converting enzyme inhibitor and corticosteroid for patients who have significant proteinuria [7, 10]. Earlier studies supported that corticosteroid was associated with the improvement in clinical outcomes [10]. As in our study, clinical outcomes including hypertension, edema, hematuria, and proteinuria improved at 12 months after treatment. Sean J. Barbour et al. [11] showed median eGFR at biopsy was 98 ml/min per 1.73 m^2^ with decreased proteinuria. The progression of IgA nephropathy was typically slow. In our study, 24% of patients developed chronic kidney disease in 2 years from diagnosis. One study showed that 10–13% of children will reach end-stage renal disease(ESRD), and within 20 years, 20–30% would have ESRD [8].

The clinicopathologic correlation in IgA nephropathy especially with MEST-C scores becomes a useful information to predict renal outcomes and for effective management decisions. This study found that mesangial hypercellularity and segmental glomerulosclerosis were frequent renal pathological findings in children with IgA nephropathy. The study from children also reported the similar results that segmental sclerosis/adhesion lesion (62%) and mesangial proliferation (45%), were found as major findings [4]. These results were in contrast to adult patients, from the Oxford classification study that had shown the amount of endocapillary hypercellularity was more frequent, whereas tubular atrophy/interstitial fibrosis was less frequent among pediatric patients compared to adults [12].

The variation in clinical symptoms of IgA nephropathy can provide a clue for renal pathology. Oxford classification 2016 [3] has been a reference point in our study. Our study showed that microscopic hematuria was strongly associated with mesangial hypercellularity which was indicated as an acute glomerular lesion. Moreover, hypertension is a prognostic factor for segmental glomerulosclerosis (S score). Similar result was also shown in previous study that the S score was associated with reduced eGFR and was higher MAP at the time of biopsy [13]. Severe pathological lesions (e.g., S, T, C) were associated with lower eGFR, higher blood pressure, and higher proteinuria, that were consistent with other findings. Glomerular hypertension may mediate progressive renal damage by leading to glomerular hyperfiltration and glomerular enlargement [14]. The previous study showed S lesion was associated with more proteinuria at presentation and more rapid decline in renal function [4]. In our study, tubular atrophy/interstitial fibrosis, and crescents lesions showed significant association with nephrotic range proteinuria in univariate Cox analysis, but it failed to attain independent significance in a multivariate model. As well as the crescent lesion was significantly associated with rapidly progressive glomerulonephritis and hypertension in univariate Cox analysis, but it was not in a multivariate model.

The presence of M1 or S1 was a histological marker predicting the benefits of steroid therapy [15]. In our study, more than half of patients that mostly had M1 and S1 scores on renal pathology received corticosteroids. After treatment follow-up, the mean serum creatinine and eGFR had not changed. In pediatric IgA nephropathy, the attempts to validate the value of MEST-C scores have always faced the problems of too few endpoints (50% decline in eGFR or ESRD) in cohorts with only a few hundred cases, and a median follow-up of 5–10 years, that is insufficient to detect a functional decline in cases with early diagnosis. As expected, T lesions are the strongest risk factors for progression in children as well as in adults, but using only a T score for selecting the treatment may be only a caution in aggressive therapy when fibrotic changes are too extensive [16]. We also found a strong correlation between T score and immunosuppressive drug use. The intensive treatment was used more in the patients with tubular atrophy/interstitial fibrosis lesion on renal biopsy than other lesions from MEST-C scores.

The presentation of crescent formation correlated with using an immunosuppressive drug such as cyclophosphamide [3]. Our study showed a similar result that pulse methylprednisolone and cyclophosphamide were used significantly more in patients with crescentic lesions than in other lesions.

The previous study has mentioned that the E lesion was not predictive of the outcome but associated with treatment. Presenting E1 were more likely to receive immunosuppressive therapy [4], most frequently corticosteroids [3]. This contrasts with our study that E lesion alone was not related to immediate treatment. In fact, the significant correlation with the treatment decisions in the short-term outcomes among the study population were mainly found in T and C lesions.

## Conclusions

In summary, the most common clinical manifestations are microscopic hematuria and proteinuria. Mesangial hypercellularity was the most common pathological finding associated with microscopic hematuria and segmental glomerulosclerosis that was associated with hypertension. The intensive treatment was used more in the patients with tubular atrophy/interstitial fibrosis lesions. The correlation between the presenting of symptoms and the severity of pathological findings influenced the treatment decisions in the short-term outcomes.

## Data Availability

All data generated or analyzed during this study are included in this published article.
